# Single-cell RNA sequencing reveals tumor immune microenvironment in human hypopharygeal squamous cell carcinoma and lymphatic metastasis

**DOI:** 10.3389/fimmu.2023.1168191

**Published:** 2023-07-12

**Authors:** Ce Li, Rui Guan, Wenming Li, Dongmin Wei, Shengda Cao, Chenyang Xu, Fen Chang, Pin Wang, Long Chen, Dapeng Lei

**Affiliations:** Department of Otorhinolaryngology, Qilu Hospital of Shandong University, NHC Key Laboratory of Otorhinolaryngology (Shandong University), Jinan, Shandong, China

**Keywords:** single-cell RNA sequencing, hypopharygeal squamous cell carcinoma, tumor immune microenvironment, IGHA1, IGHG1

## Abstract

**Background:**

Human hypopharygeal squamous cell carcinoma (HSCC) is a common head and neck cancer with a poor prognosis in advanced stages. The occurrence and development of tumor is the result of mutual influence and co-evolution between tumor cells and tumor microenvironment (TME). Tumor immune microenvironment (TIME) refers to the immune microenvironment surrounding tumor cells. Studying TIME in HSCC could provide new targets and therapeutic strategies for HSCC.

**Methods:**

We performed single-cell RNA sequencing (scRNA-seq) and analysis of hypopharyngeal carcinoma, paracancerous, and lymphoid tissues from five HSCC patients. Subdivide of B cells, T cells, macrophages cells, and monocytes and their distribution in three kinds of tissues as well as marker genes were analyzed. Different genes of IGHG1 plasma cells and SPP1+ macrophages between HSCC tissues, adjacent normal tissues and lymphatic tissues were analyzed. Additionally, we studied proliferating lymphocytes, T cells exhaustion, and T cell receptor (TCR) repertoire in three kinds of tissues.

**Results:**

Transcriptome profiles of 132,869 single cells were obtained and grouped into seven cell clusters, including epithelial cells, lymphocytes, mononuclear phagocytics system (MPs), fibroblasts, endothelial cells (ECs), plasmacytoid dendritic cells (pDCs), and mast cells. Tumor metastasis occurred in three lymphoid tissues. Four distinct populations were identified from lymphocytes, including B cells, plasma cells, T cells and proliferating lymphocytes. We found IGHA1 and IGHG1 specific plasma cells significantly overexpressed in HSCC tissues compared with normal hypopharygeal tissues and lymphatic tissues. Five distinct populations from MPs were identified, including macrophages, monocytes, mature dendritic cells (DCs), Type 1 conventional dendritic cells (cDC1) and Type 2 conventional dendritic cells (cDC2). SPP1+ macrophages were significantly overexpressed in HSCC tissues and lymphatic tissues compared with normal hypopharygeal tissues, which are thought to be M2-type macrophages. Exhaustion of CD8+ Teff cells occurred in HSCC tissues. At last, we verified that IgA and IgG1 protein expression levels were significantly up-regulated in HSCC tissues compared to adjacent normal tissues.

**Conclusion:**

Overall, this study revealed TIME in HSCC and lymphatic metastasis, and provided potential therapeutic targets for HSCC.

## Introduction

Hypopharyngeal cancer accounts for 3% to 5% of head and neck squamous cell carcinomas (1). The vast majority of hypopharyngeal cancers are hypopharyngeal squamous cell carcinomas (HSCC) (2). Patients with HSCC are predominantly male and usually have a history of smoking or heavy alcohol consumption (3). Hypopharyngeal has the worst prognosis of all head and neck cancers, with a reported 5-year overall survival rate of 30% to 35% (4). Hypopharyngeal cancer is usually detected late, with 70% to 85% of cases diagnosed at stage III or IV, approximately 60% to 80% of patients have ipsilateral cervical lymph node metastases (5), and 40% have contralateral cervical occult lymph node metastases (6). Relapse is fairly common, with nearly 50% of patients relapse within one year of diagnosis, often with distant metastases diagnosed (7). For patients with locally advanced hypopharyngeal carcinoma, surgery combined with chemoradiotherapy can significantly improve local control rates and systemic efficacy (8). Although the application of emerging therapeutic approaches such as immunotherapy and targeted therapy have greatly improved the prognosis of cancer patients, the survival of HSCC patients has not been significantly prolonged (9).

Single-cell transcriptome sequencing (scRNA-seq) technology is a technology which isolates single cells from cell populations in tissue or body fluid samples, obtains relevant data through unbiased, high-throughput and high-resolution transcriptome sequencing, and finally performs informative analysis (10). ScRNA-seq can not only reveal the unique changes of each cell, but also discover completely new cell types (11). Single-cell genome sequencing can be applied to detect genomic stability and genomic variation, thus providing new insights into human understanding of the physiological and pathological functions of cells (12). ScRNA-seq technology provides a powerful new way to characterize the clonal diversity of tumor cells and explore the role of rare cells in tumor development (13). ScRNA-seq has been used in a variety of human tumors, including glioma (14), lung cancer (15), hepatocellular carcinoma (16), nasopharyngeal carcinoma (17), colorectal cancer (18), ovarian cancer (19), gallbladder cancer (20), oral cancer (21), laryngeal carcinoma (22), etc. Chen et al. constructed the first single cell transcriptome map of hypopharyngeal cancer, revealed the complex crosstalk in HSCC and found BMPR promotes HSCC cells proliferation and migration (23).

The tumor microenvironment (TME) refers to the special environment in which tumor cells grow by interacting with the extracellular matrix during the growth process (24). Tumor immune environment (TIME) is an important part of TME, including tumor-associated macrophages (TAM), mast cells, T lymphocytes, B lymphocytes, and natural killer (NK) cells, myeloid-derived suppressor cells (MDSC), and other subgroups (25). Single-cell RNA sequencing can accurately identify different immune cell populations in the microenvironment based on the single-cell level and biomarkers that can be used to characterize such cells, providing the cellular composition and distribution characteristics of the tumor immune microenvironment from a holistic perspective, thereby revealing their functional states and discovering potential immunotherapy targets (26). Studying the tumor immune microenvironment by using single-cell sequencing has been applied to melanoma (27), liver cancer (28), lung cancer (29), breast cancer (30), colorectal cancer (31) and so on. However, the immune microenvironment in HSCC has not been explored.

In the present study, TIME in HSCC and lymphatic metastasis was revealed for the first time by performing scRNA-seq in hypopharyngeal carcinoma, paracancerous, and lymphoid tissues from HSCC patients, which may improve our current understanding of the mechanisms of HSCC development and progression.

## Materials and methods

### Sample collection and processing

HSCC tissues, adjacent normal tissues and lymph tissues from five HSCC patients were collected and stored at -20°C. All patient data used in the study were approved by the Ethics Committee of Qilu Hospital of Shandong University. The patients participating in the program were informed.

### Tissue dissociation and preparation

Fresh tumor tissue was stored in GEXSCOPE^®^ tissue preservation solution (Singleron) and shipped on ice to the Singleron laboratory as soon as possible. Samples were washed 3 times with Hanks’ Balanced Salt Solution (HBSS) and cut into 1-2 mm pieces. The tissue sections were then digested with 2 ml of GEXSCOPE^®^ tissue dissociation solution (Singleron) and placed in 15 ml centrifuge tubes at 37°C with continuous agitation for 15 minutes. After digestion, the samples were filtered through a 40 μm sterile filter, centrifuged at 1000 RPM for 5 min, the supernatant was discarded, and the pellet was resuspended in 1 ml of PBS (HyClone). To remove erythrocytes, 2 ml of GEXSCOPE^®^ erythrocyte lysis buffer (Singleron) was added for 10 minutes at 25°C. Centrifuge at 500 × g for 5 min and suspend in PBS. Samples were stained with trypan blue (Sigma) and evaluated under a microscope. Cell activity and cell concentration were measured using a fluorescence cell analyzer (Countstar Rigel S2). If there were a lot of dead cells or debris, Biotec Dead Cell Removol Kit (Miltenyi Biotec) were used for removal.

### Single cell RNA sequencing

A single-cell suspension was prepared at a concentration of 1 × 10^5^ cells/mL in PBS (HyClone). The scRNA-Seq library was constructed by the GEXSCOPE^®^ Single Cell RNA Library Kit (Singleron Biotechnologies) according to the Singleron GEXSCOPE^®^ protocol (32). Individual libraries were diluted to 4 nM and pooled for sequencing. 150 bp paired-end sequencing was performed using Illumina HiSeq X.

### ScRNA-seq quantifications and statistical analysis

The batch-effect was assessed and corrected using the Harmony algorithm. Raw reads are processed through an internal pipeline to generate gene expression profiles. Briefly, cell barcodes and UMIs were extracted after filtering reads in the absence of multiple t-tails. Read 2 will be trimmed with splicing and poly A tails (FASTP V1) and then aligned to GRCh38 using integrated version 92 gene annotation (FASTP 2.5.3A and featurecount 1.6.2) (33). Reads for the same cell barcode, UMI, and gene were combined to calculate the number of UMIs per gene per cell. The UMI count table for each cell barcode was used for further analysis. Cell type identification and cluster analysis were performed using the Seurat program (34, 35). The RNA sequencing data were analyzed using the Seurat program (http://satijalab.org/seurat/, R package, v. 3.0.1). Loaded the UMI count table into R using read table function, then set the parameter resolution of the FindClusters function to 0.6 for cluster analysis. Used the findmarker function to identify differentially expressed genes (DEGs) between different samples or consecutive clusters. GO function enrichment analysis was performed on gene sets using ClusterProfiler software to find biological functions or pathways significantly associated with specific expressed genes (36).

### T cell receptor library preparation and scRNA-seq

The single cell suspension (1×10^5^ cells/mL) was loaded into microfluidic devices. Subsequently, the scTCR-seq libraries were constructed according to the protocol of GEXSCOPE Single Cell Immuno-TCR Kit (Singleron Biotechnologies). In brief, the magnetic beads with molecular labels captured the poly (A) tail and the T-cell receptor (TCR) region on the mRNA to label the cells and mRNA after the cells were lysed. Afterwards, the magnetic beads in the chip were collected and then mRNAs captured by the magnetic beads were reverse transcribed into complementary DNA (cDNA) and amplified. Sequencing libraries suitable for the Illumina sequencing platform were constructed after partial cDNA fragmentation and splicing. The remaining cDNA was enriched for the immune receptor (TCR), and the enriched products were amplified by PCR to construct a sequencing library suitable for the Illumina sequencing platform. Finally, each library was sequenced on Illumina HiSeq X with 150 bp paired-end reads.

### TCR library analysis

TCR clonotypes assignment was performed using Cell Ranger (v4.0.0) vdj pipeline with GRCh38 as reference. In brief, a TCR diversity metric was obtained, which contains the frequency of clonotype and barcode information. Only cells with one productive TCR α-chain (TRA) and one productive TCR β-chain (TRB) were kept for further analysis. Each unique TRA(s)-TRB(s) pair was defined as a clonotype. If one clonotype was present in at least two cells, cells harboring this clonotype were considered to be clonal and the number of cells with such pairs indicated the degree of clonality of the clonotype. The TCR diversity index was calculated using the vegan package in R, with the shanno and invsimpson indices being computed through the diversity() function, while Chao and ACE were calculated using the estimateR() function. After computing the diversity for each sample based on the frequency of different clonotypes, plotted the boxplot by R package ggplot2.

### Primary analysis of raw read data

The raw reads were processed to remove low quality using fastQC v0.11.4 (https://www.bioinformatics.babraham.ac.uk/projects/fastqc/) (37) and fastp (38). Poly-A tail and linker sequences were removed by cutadapt (39). After quality control, the reads were mapped to the reference genome GRCh38 (integrated version 92 annotation) using STAR v2.5.3 (40). Gene counts and UMI counts were obtained by using featureCounts v1.6.2 software (41). Expression matrix files were generated from gene counts and UMI counts for subsequent analysis.

### Quality control, dimension-reduction and clustering

Cells with gene counts less than 200 or in the top 2% were excluded, and cells with UMI counts in the top 2% were excluded. Removed cells containing more than 20% mitochondria. Used functions in Seurat V3.1.2 for reduction and clustering (42). All gene expressions were normalized and scaled using NormalizeData() and ScaleData(). FindVariableFautres() selects the top 2000 variable genes for PCA analysis. FindClusters() divides the cells into 31 clusters using the top 20 principal components and a resolution parameter of 1.2. For subclustering of seven cell types, set the resolution to 0.8. Applied the Uniform Manifold Approximationand Projection (UMAP) algorithm to visualize cells in two-dimensional space.

### Differentially expressed genes analysis

Seurat FindMarkers() selected genes expressed by more than 10% of the cells in the cluster with an average log (fold change) greater than 0.25 as DEGs based on the Wilcox likelihood ratio test with default parameters.

### Cell type annotation

The cell type identity of each cluster was determined by the expression of canonical markers found in DEGs combined with the knowledge of the published literature (43). Seurat DoHeatmap()/DotPlot()/Vlnplot() generated a heatmap/dotplot/violin plot showing the markers used to identify each cell type.

### Pathway enrichment analysis

To investigate the potential functions of subdivided cells, Gene Ontology (GO) and Kyoto Encyclopedia of Genes and Genomes (KEGG) analyses were used with the “clusterProfiler” R package version (36). Pathways with P_ADJ values less than 0.05 were considered significantly enriched. For GSVA pathway enrichment analysis, the average gene expression per cell type was used as input data using the GSVA software package (44). Reference Gene Ontology genes include Molecular Function (MF), Biological Process (BP), and Cellular Component (CC) categories. The protein-protein interactions (PPIs) of DEGs in different clusters were predicted based on the interactions of known genes with associated GO terms in StringDB (1.22.0) (45).

### Trajectory analysis

To map the differentiation and transformation of cell subtypes in HSCC tissues, adjacent normal tissues, and lymphatic metastatic tissues, a spurious time trajectory analysis was performed using Monocle2 (46). To construct trajectories, using differentially expressed genes to rank cells in order of spatiotemporal differentiation, we performed FindVairable Features and dimensionality reduction by using DDRTree. Trajectories were visualized by plot_cell_trajectory().

### RNA velocity

For RNA velocity, BAM files containing tumor or epithelial cells and the reference genome GRCh38 were used for analysis in Python with velocyto (v 0.2.3) and scVelo (v 0.17.17), default parameters. The results were projected onto the UMAP map from the Seurat cluster analysis for visual consistency.

### Expression programs analysis

Transcription programs were extracted using the cNMF algorithm, the top 50 genes were used as meta-signatures, and the scores for each program in each cell were calculated based on the meta-signatures. The metaprogram performed computations and hierarchical clustering based on artificial correlations between each program.

### UCell gene set scoring

Gene set scoring Gene set scoring was performed using the R package UCell v 1.1.0 (47). UCell scores are based on the Mann-Whitney U statistic by ranking query genes’ in order of their expression levels in individual cells. Because UCell is a rank-based scoring method, it is suitable to be used in large datasets containing multiple samples and batches.

### Immunohistochemistry staining and statistical analysis

Tissue sections were fixed, dehydrated, and antigenically repaired. At first, the first antibody, a specific antibody that recognizes the antigen was added, and then, the second antibody, a biotin-labeled antibody that recognizes the FC segment of the first antibody was added. After that, the lecitin, biotin, and horseradish peroxidase complex was added, which was generally configured in the first 30 minutes and finally displayed with the substrate of the enzyme. At last, Observed and photographed by using a microscope. IgA antibody (ImmunoWay, YT2281, 1:200) and IgG1 antibody (ImmunoWay, YT2293, 1:200) were used for IHC staining. SPSS 22.0 statistical software package was used for statistical analysis. The two-tailed Student's t test was used to assess the statistical differences between the groups. The data were consistent with normal distribution, and P<0.05 was considered statistically significant.

## Results

### Single-cell RNA expression profiling in HSCC

**Figure 1:**
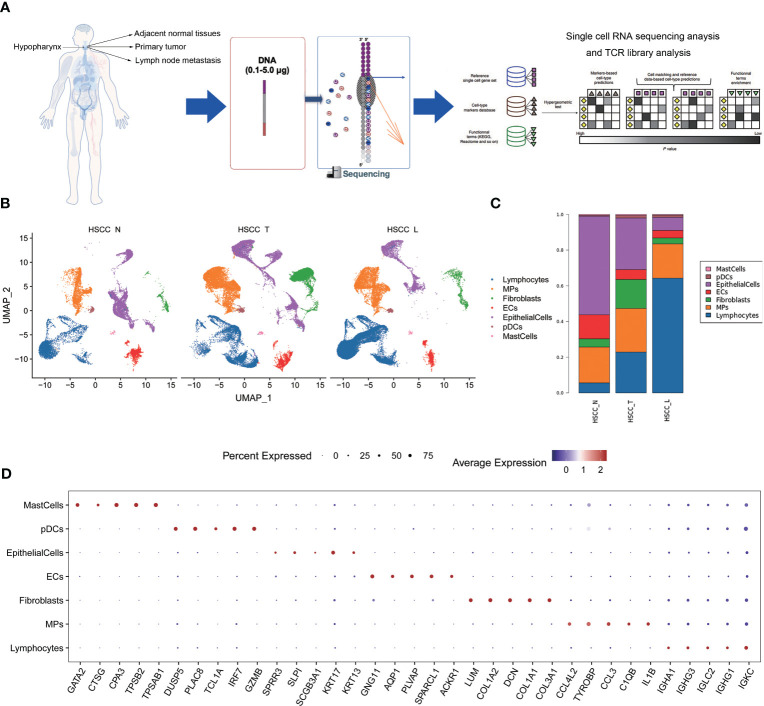
Distinct cell types in HSCC tissues, adjacent normal tissues and lymphatic tissues identified through integrating single-cell transcriptomic data. **(A)** Overview of the study design, sample collection, single cell preparation, sequencing, and bioinformatic analysis. **(B)** Uniform manifold approximation and projection (UMAP) plot showing the clustering of different cell subsets in HSCC tissues, adjacent normal tissues, and lymphatic tissues. **(C)** Sample cell composition histogram of HSCC tissues, adjacent normal tissues, and lymphatic tissues. **(D)** Bubble chart of top 5 differential genes in each cell subset.

To explore the cellular diversity and microenvironment composition of HSCC, we performed scRNA-seq and T cell receptor (TCR) analysis of primary HSCC, adjacent normal, and lymphoid tissues from five HSCC patients (**Figure 1A**). After quality control assessment, we obtained transcriptomes of 132,869 single cells using the Singleron™ Single-Cell mRNA Whole Transcriptome Analysis System, of which 52,145 cells were derived from primary tumor tissue, 39,757 cells were derived from lymph tissue cells, and 40,967 cells were derived from adjacent normal tissue. Tumor metastasis occurred in three lymphoid tissues while it did not occur in two lymphoid tissues. Seven distinct cell populations were identified from whole single-cell analysis based on t-distributed stochastic neighbor embedding (t-SNE) analysis and canonical marker expression, including lymphocytes, MPs, fibroblast cells, ECs, epithelial cells, pDCs and mast cells (**Figure 1B**). These cell populations were unevenly distributed in different kinds of tissues (**Figures 1C**; **S1A**). The proportion of lymphocytes was highest in lymphoid tissues, followed by HSCC tissue, and lowest in normal hypopharyngeal tissue. The proportion of MPs was highest in HSCC tissues. A dot plot of the top 5 differential genes in each cell subset were shown (**Figure 1D**). These results reveal the different cell types distribution in normal hypopharyngeal tissues, HSCC tissue and tumor metastasis tissues.

### Subpopulations and transcriptome landscape of lymphocytes and MPs in HSCC and lymphoid tissues

**Figure 2:**
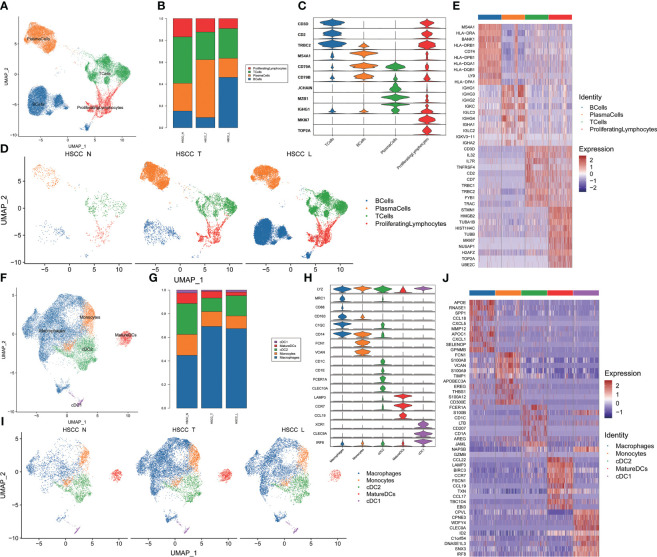
Subpopulations and transcriptome landscape of lymphocytes and MPs in HSCC tissues, adjacent normal tissues, and lymphatic tissues. **(A)** Uniform manifold approximation and projection (UMAP) plot showing the sub classification of lymphocytes. **(B)** Bar charts showing the proportion of each lymphocytes subtype in HSCC tissues, adjacent normal tissues, and lymphatic tissues. **(C)** Stacked vin plot of marker genes in each cell subset. **(D)** UMAP plot of four subtypes colored in HSCC tissues, adjacent normal tissues, and lymphatic tissues. **(E)** Heatmap showing the top 10 marker genes of each subpopulation. **(F)** Uniform manifold approximation and projection (UMAP) plot showing the sub classification of MPs. **(G)** Bar charts showing the proportion of each MPs subtype in HSCC tissues, adjacent normal tissues, and lymphatic tissues. **(H)** Stacked vin plot of marker genes in each cell subset. **(I)** UMAP plot of five subtypes colored in HSCC tissues, adjacent normal tissues, and lymphatic tissues. **(J)** Heatmap showing the top 10 marker genes of each subpopulation.

We then discovered the role of immune cells in HSCC, which mainly lymphocytes and MPs. Four distinct populations were identified from lymphocytes, including B cells, plasma cells, T cells and proliferation lymphocytes (**Figure 2A**). These cell populations distributed differently in different tissues (**Figures 2B**, **S1B**). The stacked vin plot showed marker genes in each cell subset (**Figure 2C**). UMAP plot showed six subtypes colored in HSCC tissues, adjacent normal tissues and lymphatic tissues (**Figure 2D**). The heatmap of the top10 differential genes in each cell subset was shown (**Figure 2E**). Five distinct populations were identified from MPs, including macrophages, monocytes, mature dendritic cells (DCs), cDC1 and cDC2 (**Figure 2F**). These cell populations distributed differently in different tissues (**Figures 2G**, **S1C**). Stacked vin plot showed marker genes in each cell subset (**Figure 2H**). UMAP plot showed five subtypes colored in HSCC tissues, adjacent normal tissues and lymphatic tissues (**Figure 2I**). The heatmap of top10 differential genes in each cell subset was shown (**Figure 2J**).

### Subpopulations and transcriptome landscape of B cells and T cells in HSCC and lymphoid tissues

**Figure 3:**
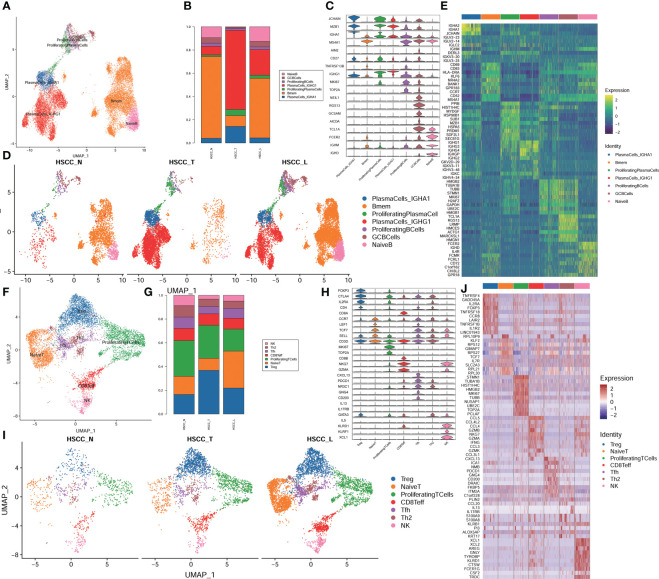
Subpopulations and transcriptome landscape of B cells and T cells in HSCC tissues, adjacent normal tissues, and lymphatic tissues. **(A)** Uniform manifold approximation and projection (UMAP) plot showing the sub classification of B cells. **(B)** Bar charts showing the proportion of each B cells subtype in HSCC tissues, adjacent normal tissues, and lymphatic tissues. **(C)** Stacked vin plot of marker genes in each cell subset. **(D)** UMAP plot of seven subtypes colored in HSCC tissues, adjacent normal tissues, and lymphatic tissues. **(E)** Heatmap showing the top 10 marker genes of each subpopulation. **(F)** Uniform manifold approximation and projection (UMAP) plot showing the sub classification of T cells. **(G)** Bar charts showing the proportion of each T cells subtype in HSCC tissues, adjacent normal tissues, and lymphatic tissues. **(H)** Stacked vin plot of marker genes in each cell subset. **(I)** UMAP plot of seven subtypes colored in HSCC tissues, adjacent normal tissues, and lymphatic tissues. **(J)** Heatmap showing the top 10 marker genes of each subpopulation.

**Figure 4:**
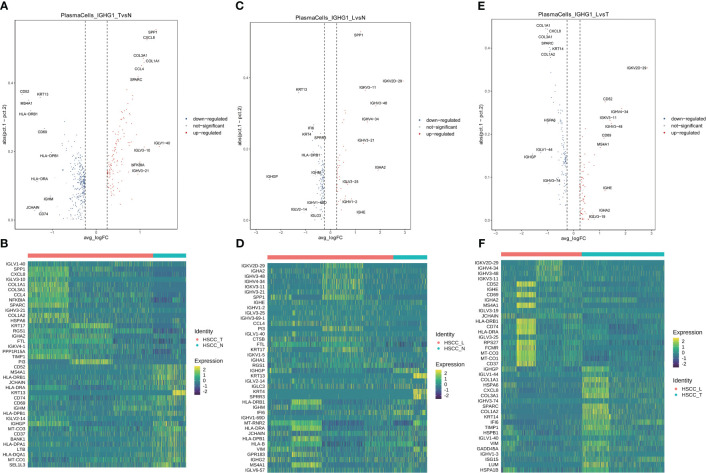
Volcano plots and heatmaps showing different genes of IGHG1 plasma cells in HSCC tissues, adjacent normal tissues, and lymphatic tissues. **(A)** Volcano plot showing different genes of IGHG1 plasma cells between HSCC tissues and adjacent normal tissues. **(B)** Heatmap showing different genes of IGHG1 plasma cells between HSCC tissues and adjacent normal tissues. **(C)** Volcano plot showing different genes of IGHG1 plasma cells between lymphatic tissues and normal hypopharynx tissues. **(D)** Heatmap showing different genes of IGHG1 plasma cells between lymphatic tissues and normal hypopharynx tissues. **(E)** Volcano plot showing different genes of IGHG1 plasma cells between lymphatic tissues and HSCC tissues. **(F)** Heatmap showing different genes of IGHG1 plasma cells between lymphatic tissues and HSCC tissues.

Seven distinct populations were identified from B cells, including naive B cells, GC B cells, proliferating B cells, proliferating B cells IGHG1 plasma cells, IGHA1 plasma cells, and Bmem cells (**Figures 3A**). These cell populations distributed differently in different tissues (**Figures 3B**, **S1D**). IGHA1 and IGHG1 specific plasma cells were significantly overexpressed in HSCC tissues compared with normal hypopharygeal tissues. Stacked vin plot showed marker genes in each cell subset (**Figure 3C**). UMAP plot showed seven subtypes colored in HSCC tissues, adjacent normal tissues and lymphatic tissues (**Figure 3D**). The heatmap of the top10 differential genes in each cell subset was shown (**Figure 3E**). Seven distinct populations were identified from T cells, including NK, Th2, Tfh, CD8Teff, Proliferating T cells, Naive T, and Treg cells (**Figures 3F**). These cell populations distributed differently in different tissues (**Figures 3G**, **S1E**). Treg cells were significantly overexpressed in HSCC tissues and lymphatic tissues compared with normal hypopharygeal tissues. Naive T cells were significantly overexpressed in lymphatic tissues compared with normal hypopharygeal tissues and HSCC tissues. Stacked vin plot showed marker genes in each cell subset (**Figure 3H**). UMAP plot showed seven subtypes colored in HSCC tissues, adjacent normal tissues and lymphatic tissues (**Figure 3I**). The heatmap of the top10 differential genes in each cell subset was shown (**Figure 3J**). The volcano plots and the heatmaps showed different genes of IGHG1 plasma cells between HSCC tissues, adjacent normal tissues, and lymphatic tissues. IGLV1-40, SPP1, CXCL8, IGLV3-10, and COL1A1 were the most up-regulated genes in HSCC tissues compared with adjacent normal hypopharynx tissues (**Figures 4A, B**). IGKV2D-29, IGHA2, IGHV3-48, IGHV4-34, and IGKV3-11 were the most up-regulated genes in lymphatic tissues compared with adjacent normal hypopharynx tissues (**Figures 4C, D**). IGKV2D-29, IGHV4-34, IGHV3-48, IGKV3-11, and CD52 were the most up-regulated genes in lymphatic tissues compared with HSCC tissues (**Figures 4E, F**).

### Subpopulations, pseudotime trajectory and transcriptome landscape of macrophages and monocytes in HSCC and lymphoid tissues

**Figure 5:**
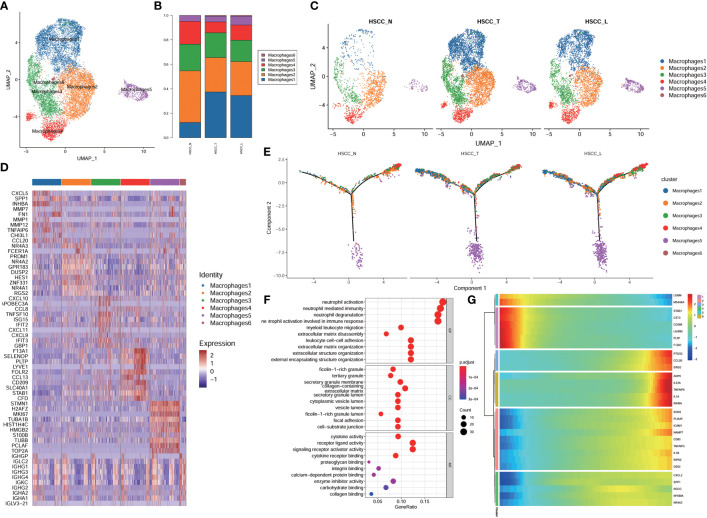
Subpopulations, pseudotime trajectory and transcriptome landscape of macrophages in HSCC tissues, adjacent normal tissues, and lymphatic tissues. **(A)** Uniform manifold approximation and projection (UMAP) plot showing the sub classification of macrophages. **(B)** Bar charts showing the proportion of each macrophages subtype in HSCC tissues, adjacent normal tissues, and lymphatic tissues. **(C)** UMAP plot of six subtypes colored in HSCC tissues, adjacent normal tissues, and lymphatic tissues. **(D)** Heatmap showing the top 10 marker genes of each subpopulation. **(E)** Pseudotemporal trajectory of six macrophages cell types in all tissues. **(F)** Upregulated GO pathway in macrophages1. **(G)** Heatmap for clustering the top genes that affected cell fate decisions.

**Figure 6:**
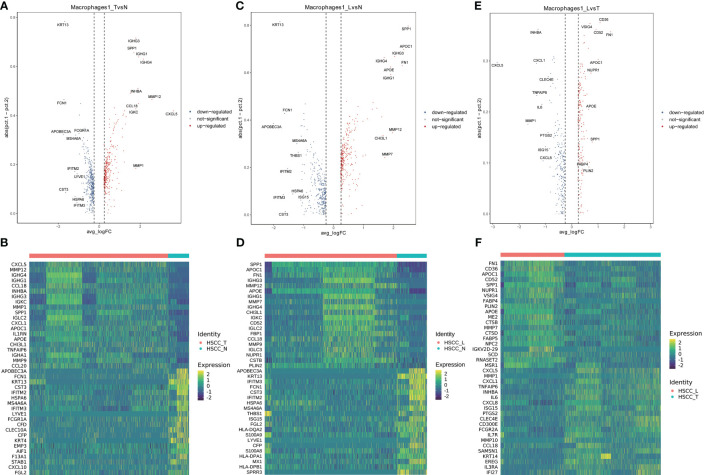
Volcano plots and heatmaps showing different genes of macrophage1 in HSCC tissues, adjacent normal tissues, and lymphatic tissues. **(A)** Volcano plot showing different genes of macrophage1 between HSCC tissues and adjacent normal tissues. **(B)** Heatmap showing different genes of macrophage1 between HSCC tissues and adjacent normal tissues. **(C)** Volcano plot showing different genes of macrophage1 between lymphatic tissues and normal hypopharynx tissues. **(D)** Heatmap showing different genes of macrophage1 between lymphatic tissues and normal hypopharynx tissues. **(E)** Volcano plot showing different genes of macrophage1 between lymphatic tissues and HSCC tissues. **(F)** Heatmap showing different genes of macrophage1 between lymphatic tissues and HSCC tissues.

Six distinct populations were identified from macrophages cells (**Figure 5A**). These cell populations distributed differently in different tissues (**Figures 5B**, **S1F**). Macrophage1 was significantly overexpressed in HSCC tissues and lymphatic tissues compared with normal hypopharygeal tissues, with marker genes including CXCL5, SPP1, INHBA, MMP1, FN1, MMP12, TNFAIP6, CHI3L1 and CCL20. UMAP plot showed six subtypes colored in HSCC tissues, adjacent normal tissues and lymphatic tissues (**Figure 5C**). Macrophage1 can also be called SPP1+ macrophages according to its marker genes. Heatmap of the top10 differential genes in each cell subset was shown (**Figure 5D**). Pseudo-time of macrophages expression profiles was reconstituted (**Figure 5E**). Molecular functions, cytological components, biological and KEGG pathways of macrophages1 were shown (**Figures 5E, F**). We also detected the activation of multiple key regulators and TFs in six macrophages populations (**Figure 5G**). The heatmaps and the volcano plots showed different genes of macrophage1 cells between HSCC tissues, adjacent normal tissues, and lymphatic tissues. CXCL5, MMP12, IGHG4, IGHG1, and CCL18 were the most up-regulated genes in HSCC tissues compared with adjacent normal hypopharynx tissues (**Figures 6A, B**). SPP1, APOC1, FN1, IGHG3, and MMP12 were the most up-regulated genes in lymphatic tissues compared with adjacent normal hypopharynx tissues (**Figures 6C, D**). FN1, CD36, APOC1, CD52, and SPP1 were the most up-regulated genes in lymphatic tissues compared with HSCC tissues (**Figures 6E, F**). Four distinct monocytes populations were identified from monocytes cells (**Figure S2A**). These cell populations distributed differently in different tissues (**Figures S1G, S2B**). UMAP plot showed four subtypes colored in HSCC tissues, adjacent normal tissues and lymphatic tissues (**Figure S2C**). Dotplot of the top5 differential genes and heatmap of the top10 differential genes in each cell subset were shown (**Figures S2D, E**).

### Subpopulations and transcriptome landscape of proliferating lymphocytes and T cell exhaustion in HSCC and lymphoid tissues

**Figure 7:**
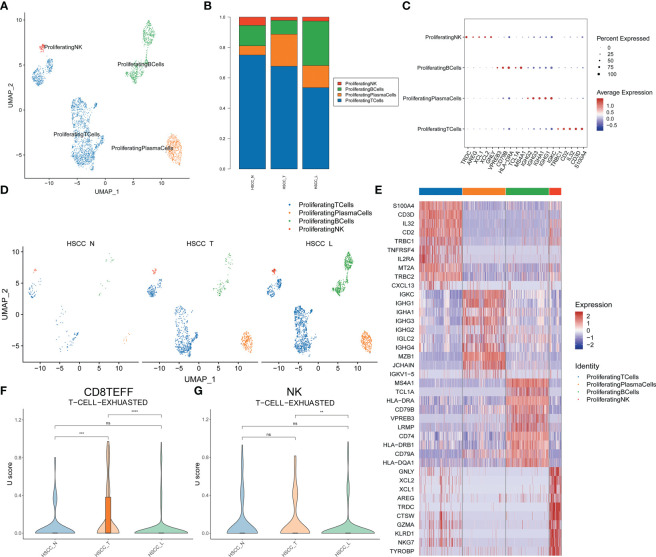
Subpopulations and transcriptome landscape of proliferating lymphocytes and cell exhausted levels in HSCC tissues, adjacent normal tissues, and lymphatic tissues. **(A)** Uniform manifold approximation and projection (UMAP) plot showing the sub classification of proliferating lymphocytes. **(B)** Bar charts showing the proportion of each proliferating lymphocytes subtype in HSCC tissues, adjacent normal tissues, and lymphatic tissues. **(C)** Bubble chart showing 5 typical genes expressed in each subtype. **(D)** UMAP plot of four subtypes colored in HSCC tissues, adjacent normal tissues, and lymphatic tissues. **(E)** Heatmap showing the top 10 marker genes of each subpopulation. **(F)** Exhausted effector CD8 T cells levels in HSCC tissues, adjacent normal tissues, and lymphatic tissues. **(G)** Exhausted NK T cells levels in HSCC tissues, adjacent normal tissues, and lymphatic tissues.

Four distinct populations were identified from proliferating lymphocytes, including proliferating NK, proliferating B cells, proliferating plasma cells and proliferating T cells (**Figure 7A**). These cell populations distributed differently in different tissues (**Figures 7B**; **S1H**). Proliferating T cells and proliferating NK cells were significantly overexpressed in HSCC tissues and lymphatic tissues compared with normal hypopharygeal tissues. Proliferating B cells were significantly overexpressed in lymphatic tissues compared with HSCC tissues and normal hypopharygeal tissues. Bubble chart showing 5 typical genes expressed in each subtype (**Figure 7C**). UMAP plot showed four subtypes colored in HSCC tissues, adjacent normal tissues and lymphatic tissues (**Figure 7D**). The heatmap of the top10 differential genes in each cell subset was shown (**Figure 7E**). Exhausted CD8+ effector T (Teff) cells levels and exhausted NK T cells levels in HSCC tissues, adjacent normal tissues and lymphatic tissues were measured (**Figures 7F, G**). Exhaustion of CD8+ Teff cells occurred in HSCC tissues.

### Comprehensive analysis of the TCR repertoire in HSCC and lymphoid tissues

**Figure 8:**
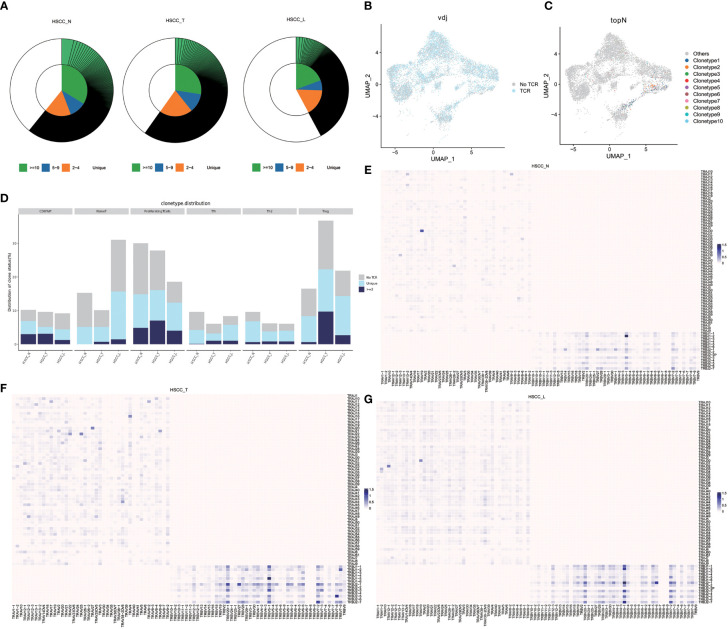
Clonal diversity and V-J pairing in HSCC tissues, adjacent normal tissues, and lymphatic tissues. **(A)** Grouped clonotype ratio circle diagram in HSCC tissues, adjacent normal tissues, and lymphatic tissues. **(B)** VDJ TCR features in HSCC tissues, adjacent normal tissues, and lymphatic tissues. **(C)** TOP10 clonotype mapping UMAP map in HSCC tissues, adjacent normal tissues, and lymphatic tissues. **(D)** Clonetypes distribution in HSCC tissues, adjacent normal tissues, and lymphatic tissues. **(E)** VJ pairs heatmap in adjacent normal tissues. **(F)** VJ pairs heatmap in HSCC tissues. **(G)** VJ pairs heatmap in lymphatic tissues.

We then studied the TCR repertoires in HSCC, adjacent normal and lymphatic tissues. The clonotype ratio showed clonal diversity of T-cell repertoires in HSCC tissues, adjacent normal tissues, and lymphatic tissues (**Figure 8A**). Clonal diversity in HSCC tissues, adjacent normal tissues, and lymphatic tissues was analyzed by using Shannon score, Inv. Simpson score, Chao score and ACE score (**Figures S3A–D**). The diversity of TCR clonal was lower in HSCC and lymphoid tissues than in normal hypopharynx tissues. VDJ TCR features and TOP10 colontypes were shown (**Figures 8B, C**). Most of TOP10 colontypes related to proliferating T cells and CD8 Teff cells. Clonotypes distribution of grouped clonotypes were analyzed (**Figure 8D**). Treg cells showed significant higher clonal expansion in HSCC tissues compared with lymphoid metastasis and normal hypopharynx tissues. VJ pairs heatmap showed different VJ rearrangement in HSCC tissues, adjacent normal tissues and lymphatic tissues (**Figures 8E–G**).

### IHC staining of IgA and IgG1 in HSCC tissues and adjacent normal tissues

**Figure 9:**
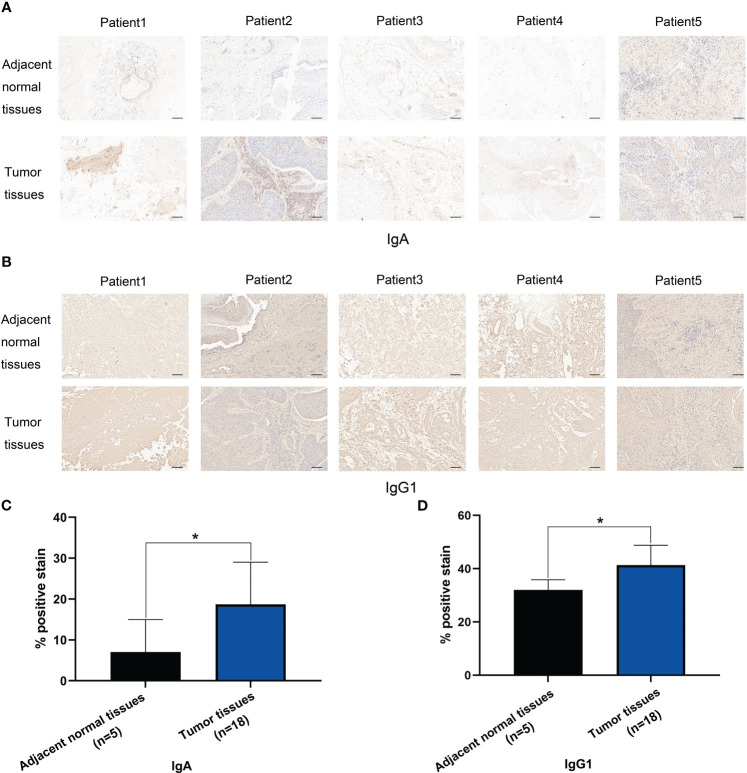
IHC stain of IgA and IgG1 in HSCC tissues and adjacent normal tissues. **(A)** IHC stain of IgA in 5 pair HSCC tissues and adjacent normal tissues. (Scale bars = 100μm). **(B)** Analysis of IgA positive stain in HSCC tissues (n=18) and adjacent normal tissues (n=5). (*, p<0.05) **(C)** IHC stain of IgG1 in 5 pair HSCC tissues and adjacent normal tissues. (Scale bars = 100μm) **(D)** Analysis of IgG1 positive stain in HSCC tissues (n=18) and adjacent normal tissues (n=5). (*, p<0.05).

At last, since IGHA2 was also upregulated in plasma cells of HSCC tissues, we verified IgA protein expression levels which encoded by IGHA1 and IGHA2 in HSCC tissues and adjacent normal tissues by using IHC staining. IgA positive stain in HSCC tissues was significantly higher than that in adjacent normal tissues (**Figures 9A, B**). We also verified IgG1 protein expression levels which encoded by IGHG1 in HSCC tissues and adjacent normal tissues by using IHC staining. IgG1 positive stain in HSCC tissues was significantly higher than that in adjacent normal tissues (**Figures 9C, D**). These results showed IgA and IgG1 may be potential diagnostic markers of HSCC.

## Discussion

HSCC is a malignant disease with a poor prognosis. Over the past few decades, TIME targeting strategies have provided new therapeutic options for cancer therapy. However, these strategies have not yet been applied clinically in HSCC because the cellular characteristics and immune microenvironment of HSCC are largely unknown. In this study, we performed single-cell transcriptomic profiling of HSCC tissues, adjacent normal tissues, and lymphoid tissues to reveal TIME in HSCC and lymphatic metastasis for the first time. These results may improve our current understanding of HSCC development and progression, and provide new therapeutic targets for HSCC.

TIME is an important component of TME, including T lymphocytes, B lymphocytes, NK cells, macrophages and other cell subsets; these immune cells not only play a role in killing tumor cells, for example, CD8+ T cells, natural killer cells, and M1 macrophages; but also promote tumor development, for example, Th2 cells, Treg cells, and M2 macrophages (48). Therefore, the tumor microenvironment has become a potential anticancer target, and its research has become a hot spot in tumor biology (49). Single-cell sequencing technology is a powerful tool for analyzing the heterogeneity of cellular components in the TME, and scRNA-seq analysis can detect more diverse TME immune cells in tumor tissues, thus serving as a highly feasible platform for analyzing the tumor microenvironment (50).

In this study, we studied the immune microenvironment in HSCC and lymphatic tissues. Seven distinct cell populations were identified from the whole single-cell analysis, including lymphocytes, MPs, fibroblasts cells, ECs, epithelial cells, pDCs, and mast cells. We focused on lymphocytes and MPs in HSCC tissues, adjacent normal tissues, and lymphatic tissues. Four distinct populations were identified from lymphocytes, including B cells, plasma cells, T cells and proliferation lymphocytes. Subdivide of B cells and T cells and their distribution in three kinds of tissues as well as marker genes were analyzed. Four distinct populations from MPs were identified, including macrophages, monocytes, cDC2, MatureDCs, and cDC1. Subdivide of macrophages cells and monocytes cells and their distribution in three kinds of tissues as well as marker genes were analyzed. We also performed TCR repertoire analysis, which including clonal diversity, clonotype distribution and V-J pairing in HSCC tissues, adjacent normal tissues, and lymphatic tissues.

We firstly verified that IGHA1 and IGHA2 up-regulated at mRNA level, and IgA up-regulated at protein level in HSCC by using single-cell sequencing and IHC staining. The basic structure of immunoglobulin is a monomer composed of four symmetrical polypeptide chains, including two identical heavy chains with larger molecular weight and two identical light chains with smaller molecular weight, and there are two light chains between the light and heavy chains. Immunoglobulin A is divided into two subclasses, IGHA1 and IGHA2, which are composed of the heavy chain α1 or α2 and the light chain respectively, and its heavy chain constant region is located on chromosome 14q32.33. IGHA1 was up-regulated and reported as an unfavorable biomarker in renal cell carcinoma (51) and prostate cancer (52), while reported as a favorable biomarker in breast cancer (53). We firstly verified that IGHG1 up-regulated at mRNA level and IgG1 up-regulated at protein level in HSCC by using single-cell sequencing and IHC staining. IGHG1 is immunoglobulin gamma-1 heavy chain constant region, belongs to immunoglobulin G (IgG), which accounts for approximately 80% of total immunoglobulins (54). Although only B cells and plasma cells are known to produce IgG, there are increasing reports suggesting that IgG can be produced by several malignant cells, such as tumor cells from the esophagus, breast, liver, or prostate (55). IGHG1 promotes tumor development in gastric cancer, breast cancer and prostate cancer *via* AKT and MEK pathway (55–57). Overall, IGHA1 and IGHG1 may be potential diagnostic markers and serum markers of HSCC.

TAMs play an important role in immunosuppressive macrophages. TAMs occupy a large proportion of tumor mesenchymal cells, most of which are migrated and differentiated from peripheral blood mononuclear cells and developed under the influence of tumor cells and their microenvironment. TAMs can secrete a variety of growth factors, cytokines, immunosuppressive mediators and proteolytic enzymes to promote tumor progression and metastasis (58). We found Macrophage1 was significantly overexpressed in HSCC tissues and lymphatic tissues compared with normal hypopharygeal tissues. Macrogphage1 may be potential tumor-associated macrophages (TAMs) in HSCC. Macrophage1 can also be called SPP1+ macrophages according to its marker genes. According to the previous literatures, the overexpression of SPP1 in macrophages is thought to correlate with the phenotype of M2-type macrophages (23). The number of M2-type macrophages was negatively correlated with progression-free survival, distant metastasis-free survival and overall survival of HNSCC patients (59). M2-type macrophages may play an important role in assisting HNSCC tumors to evade immune surveillance, and promoting tumor invasion and metastasis in HNSCC (60).

We measured exhausted CD8+ Teff cells levels and exhausted NK T cells levels in HSCC tissues, adjacent normal tissues and lymphatic tissues. We found exhaustion of CD8+ Teff occurred in HSCC tissues. CD8+ T cells exhaustion is an important factor that affecting tumor progression, its heterogeneity is closely related to the strength of immunotherapy response and survival prognosis of tumor patients (61).

In conclusion, we investigated TIME in HSCC and lymphatic metastasis by performing single-cell RNA sequencing in hypopharyngeal carcinoma, paracancerous tissues and lymphatic tissues of five HSCC patients. Different cell populations and subpopulations and their marker genes, and differentially expressed genes were identified. These results may not only elucidate the mechanism of occurrence and development of HSCC, but also provide potential therapeutic targets for HSCC.

## Data availability statement

The data presented in the study are deposited in the GEO repository, accession number GSE206038, GSE227156. The analysis of raw data will be shared on reasonable request to the corresponding author.

## Ethics statement

The studies involving human participants were reviewed and approved by Ethics Committee of Qilu Hospital of Shandong University. The patients/participants provided their written informed consent to participate in this study.

## Author contributions

DL contributed to the conception of the study; CL performed the study and wrote the manuscript; FC and PW helped in the early design. LC acquired HSCC tissues, RG, WL, DW, SC and CX helped perform the analysis with constructive discussions. All authors contributed to the article and approved the submitted version.

## References

[1] Cooper JS Porter K Mallin K Hoffman HT Weber RS Ang KK. National cancer database report on cancer of the head and neck: 10-year update. Head Neck (2009) 31:748–58. doi: 10.1002/hed.21022 19189340

[2] Chiruvella V Guddati AK. Analysis of race and gender disparities in mortality trends from patients diagnosed with nasopharyngeal, oropharyngeal and hypopharyngeal cancer from 2000 to 2017. Int J Gen Med (2021) 14:6315–23. doi: 10.2147/IJGM.S301837 PMC8495144 34629896

[3] Avincsal MO Shinomiya H Teshima M Kubo M Otsuki N Kyota N. Impact of alcohol dehydrogenase-aldehyde dehydrogenase polymorphism on clinical outcome in patients with hypopharyngeal cancer. Head Neck (2018) 40:770–7. doi: 10.1002/hed.25050 29286190

[4] Newman JR Connolly TM Illing EA Kilgore ML Locher JL Carroll WR. Survival trends in hypopharyngeal cancer: a population-based review. Laryngoscope (2015) 125:624–9. doi: 10.1002/lary.24915 25220657

[5] Gourin CG Terris DJ. Carcinoma of the hypopharynx. Surg Oncol Clin N Am (2004) 13:81–98. doi: 10.1016/S1055-3207(03)00122-4 15062363

[6] Buckley JG MacLennan K. Cervical node metastases in laryngeal and hypopharyngeal cancer: a prospective analysis of prevalence and distribution. Head Neck (2000) 22:380–5. doi: 10.1002/1097-0347(200007)22:4<380::AID-HED11>3.0.CO;2-E 10862022

[7] Hall SF Groome PA Irish J O'Sullivan B. The natural history of patients with squamous cell carcinoma of the hypopharynx. Laryngoscope (2008) 118:1362–71. doi: 10.1097/MLG.0b013e318173dc4a 18496152

[8] Garneau JC Bakst RL Miles BA. Hypopharyngeal cancer: a state of the art review. Oral Oncol (2018) 86:244–50. doi: 10.1016/j.oraloncology.2018.09.025 30409307

[9] Pai SI Faivre S Licitra L Machiels JP Vermorken JB Bruzzi P. Comparative analysis of the phase III clinical trials of anti-PD1 monotherapy in head and neck squamous cell carcinoma patients (CheckMate 141 and KEYNOTE 040). J Immunother Cancer (2019) 7:96. doi: 10.1186/s40425-019-0578-0 30944020 PMC6446400

[10] Ziegenhain C Vieth B Parekh S Reinius B Guillaumet-Adkins A Smets M. Comparative analysis of single-cell RNA sequencing methods. Mol Cell (2017) 65:631–643 e4. doi: 10.1016/j.molcel.2017.01.023 28212749

[11] Chen Z Zhou L Liu L Hou Y Xiong M Yang Y. Single-cell RNA sequencing highlights the role of inflammatory cancer-associated fibroblasts in bladder urothelial carcinoma. Nat Commun (2020) 11:5077. doi: 10.1038/s41467-020-18916-5 33033240 PMC7545162

[12] Chen C Xing D Tan L Li H Zhou G Huang L. Single-cell whole-genome analyses by linear amplification *via* transposon insertion (LIANTI). Science (2017) 356:189–94. doi: 10.1126/science.aak9787 PMC5538131 28408603

[13] Suva ML Tirosh I. Single-cell RNA sequencing in cancer: lessons learned and emerging challenges. Mol Cell (2019) 75:7–12. doi: 10.1016/j.molcel.2019.05.003 31299208

[14] Ochocka N Segit P Walentynowicz KA Wojnicki K Cyranowski S Swatler J. Single-cell RNA sequencing reveals functional heterogeneity of glioma-associated brain macrophages. Nat Commun (2021) 12:1151. doi: 10.1038/s41467-021-21407-w 33608526 PMC7895824

[15] Maynard A McCoach CE Rotow JK Harris L Haderk F Kerr DL. Therapy-induced evolution of human lung cancer revealed by single-cell RNA sequencing. Cell (2020) 182:1232–1251 e22. doi: 10.1016/j.cell.2020.07.017 32822576 PMC7484178

[16] Sun Y Wu L Zhong Y Zhou K Hou Y Wang Z. Single-cell landscape of the ecosystem in early-relapse hepatocellular carcinoma. Cell (2021) 184:404–421 e16. doi: 10.1016/j.cell.2020.11.041 33357445

[17] Gong L Kwong DL Dai W Wu P Li S Yan Q. Comprehensive single-cell sequencing reveals the stromal dynamics and tumor-specific characteristics in the microenvironment of nasopharyngeal carcinoma. Nat Commun (2021) 12:1540. doi: 10.1038/s41467-021-21795-z 33750785 PMC7943808

[18] Zhou Y Bian S Zhou X Cui Y Wang W Wen L. Single-cell multiomics sequencing reveals prevalent genomic alterations in tumor stromal cells of human colorectal cancer. Cancer Cell (2020) 38:818–828 e5. doi: 10.1016/j.ccell.2020.09.015 33096021

[19] Hu Z Artibani M Alsaadi A Wietek N Morotti M Shi T. The repertoire of serous ovarian cancer non-genetic heterogeneity revealed by single-cell sequencing of normal fallopian tube epithelial cells. Cancer Cell (2020) 37:226–242 e7. doi: 10.1016/j.ccell.2020.01.003 32049047

[20] Chen P Wang Y Li J Bo X Wang J Nan L. Diversity and intratumoral heterogeneity in human gallbladder cancer progression revealed by single-cell RNA sequencing. Clin Transl Med (2021) 11:e462. doi: 10.1002/ctm2.462 PMC8236117 34185421

[21] Puram SV Tirosh I Parikh AS Patel AP Yizhak K Gillespie S. Single-cell transcriptomic analysis of primary and metastatic tumor ecosystems in head and neck cancer. Cell (2017) 171:1611–1624 e24. doi: 10.1016/j.cell.2017.10.044 29198524 PMC5878932

[22] Song L Zhang S Yu S Ma F Wang B Zhang C. Cellular heterogeneity landscape in laryngeal squamous cell carcinoma. Int J Cancer (2020) 147:2879–90. doi: 10.1002/ijc.33192 32638385

[23] Ozato Y Kojima Y Kobayashi Y Hisamatsu Y Toshima T Yonemura Y. Spatial and single-cell transcriptomics decipher the cellular environment containing HLA-g+ cancer cells and SPP1+ macrophages in colorectal cancer. Cell Rep (2023) 42:111929. doi: 10.1016/j.celrep.2022.111929 36656712

[24] Mantovani A Romero P Palucka AK Marincola FM. Tumour immunity: effector response to tumour and role of the microenvironment. Lancet (2008) 371:771–83. doi: 10.1016/S0140-6736(08)60241-X 18275997

[25] Hanahan D Weinberg RA. Hallmarks of cancer: the next generation. Cell (2011) 144:646–74. doi: 10.1016/j.cell.2011.02.013 21376230

[26] Papalexi E Satija R. Single-cell RNA sequencing to explore immune cell heterogeneity. Nat Rev Immunol (2018) 18:35–45. doi: 10.1038/nri.2017.76 28787399

[27] Tirosh I Izar B Prakadan SM Wadsworth MH Treacy 2D Trombetta JJ. Dissecting the multicellular ecosystem of metastatic melanoma by single-cell RNA-seq. Science (2016) 352:189–96. doi: 10.1126/science.aad0501 PMC4944528 27124452

[28] Zheng C Zheng L Yoo JK Guo H Zhang Y Guo X. Landscape of infiltrating T cells in liver cancer revealed by single-cell sequencing. Cell (2017) 169:1342–1356 e16. doi: 10.1016/j.cell.2017.05.035 28622514

[29] Lavin Y Kobayashi S Leader A Amir ED Elefant N Bigenwald C. Innate immune landscape in early lung adenocarcinoma by paired single-cell analyses. Cell (2017) 169:750–765 e17. doi: 10.1016/j.cell.2017.04.014 28475900 PMC5737939

[30] Azizi E Carr AJ Plitas G Cornish AE Konopacki C Prabhakaran S. Single-cell map of diverse immune phenotypes in the breast tumor microenvironment. Cell (2018) 174:1293–1308 e36. doi: 10.1016/j.cell.2018.05.060 29961579 PMC6348010

[31] Zhang L Yu X Zheng L Zhang Y Li Y Fang Q. Lineage tracking reveals dynamic relationships of T cells in colorectal cancer. Nature (2018) 564:268–72. doi: 10.1038/s41586-018-0694-x 30479382

[32] Dura B Choi JY Zhang K Damsky W Thakral D Bosenberg M. scFTD-seq: freeze-thaw lysis based, portable approach toward highly distributed single-cell 3' mRNA profiling. Nucleic Acids Res (2019) 47:e16. doi: 10.1093/nar/gky1173 30462277 PMC6379653

[33] Liao Y Smyth GK Shi W. featureCounts: an efficient general purpose program for assigning sequence reads to genomic features. Bioinf (Oxford England) (2014) 30:923–30. doi: 10.1093/bioinformatics/btt656 24227677

[34] Satija R Farrell JA Gennert D Schier AF Regev A. Spatial reconstruction of single-cell gene expression data. Nat Biotechnol (2015) 33:495–502. doi: 10.1038/nbt.3192 25867923 PMC4430369

[35] Butler A Hoffman P Smibert P Papalexi E Satija R. Integrating single-cell transcriptomic data across different conditions, technologies, and species. Nat Biotechnol (2018) 36:411–20. doi: 10.1038/nbt.4096 PMC6700744 29608179

[36] Yu G Wang L-G Han Y Qing Q-Y. clusterProfiler: an r package for comparing biological themes among gene clusters. Omics J Integr Biol (2012) 16:284–7. doi: 10.1089/omi.2011.0118 PMC3339379 22455463

[37] Davis MPA van Dongen S Abreu-Goodger C Bartonicek N Enright AJ. Kraken: a set of tools for quality control and analysis of high-throughput sequence data. Methods (2013) 63:41–9. doi: 10.1016/j.ymeth.2013.06.027 PMC3991327 23816787

[38] Chen S Zhou Y Chen Y Gu J. Fastp: an ultra-fast all-in-one FASTQ preprocessor. Bioinformatics (2018) 34:i884–90. doi: 10.1093/bioinformatics/bty560 PMC6129281 30423086

[39] Kechin A Boyarskikh U Kel A Filipenko M. cutPrimers: a new tool for accurate cutting of primers from reads of targeted next generation sequencing. J Comput Biol (2017) 24:1138–43. doi: 10.1089/cmb.2017.0096 28715235

[40] Dobin A Davis CA Schlesinger F Drenkow J Zaleski C Jha S. STAR: ultrafast universal RNA-seq aligner. Bioinformatics (2013) 29:15–21. doi: 10.1093/bioinformatics/bts635 23104886 PMC3530905

[41] Stuart T Butler A Hoffman P Hafemeister C Papalexi E Mauck WM. Comprehensive integration of single-cell data. Cell (2019) 177:1888–1902 e21. doi: 10.1016/j.cell.2019.05.031 31178118 PMC6687398

[42] Gribov A Sill M Luck S Rucker F Dohner K Bullinger L. SEURAT: visual analytics for the integrated analysis of microarray data. BMC Med Genomics (2010) 3:21. doi: 10.1186/1755-8794-3-21 20525257 PMC2893446

[43] Zhang R Lin Y. DEG 5.0, a database of essential genes in both prokaryotes and eukaryotes. Nucleic Acids Res (2009) 37:D455–8. doi: 10.1093/nar/gkn858 PMC2686491 18974178

[44] Hanzelmann S Castelo R Guinney J. GSVA: gene set variation analysis for microarray and RNA-seq data. BMC Bioinf (2013) 14:7. doi: 10.1186/1471-2105-14-7 PMC3618321 23323831

[45] Szklarczyk D Morris JH Cook H Kuhn M Wyder S Simonovic M. The STRING database in 2017: quality-controlled protein-protein association networks, made broadly accessible. Nucleic Acids Res (2017) 45:D362–8. doi: 10.1093/nar/gkw937 PMC5210637 27924014

[46] Qiu X Hill A Packer J Lin D Ma YA Trapnell C. Single-cell mRNA quantification and differential analysis with census. Nat Methods (2017) 14:309–15. doi: 10.1038/nmeth.4150 PMC5330805 28114287

[47] Andreatta M Carmona SJ. UCell: robust and scalable single-cell gene signature scoring. Comput Struct Biotechnol J (2021) 19:3796–8. doi: 10.1016/j.csbj.2021.06.043 PMC8271111 34285779

[48] Pena-Romero AC Orenes-Pinero E. Dual effect of immune cells within tumour microenvironment: pro- and anti-tumour effects and their triggers. Cancers (2022) 14 , 48:1681. doi: 10.3390/cancers14071681 PMC8996887 35406451

[49] Lee HO Park WY. Single-cell RNA-seq unveils tumor microenvironment. BMB Rep (2017) 50:283–4. doi: 10.5483/BMBRep.2017.50.6.086 PMC5498138 28539161

[50] Kashima Y Togashi Y Fukuoka S Kamada T Irie T Suzuki A. Potentiality of multiple modalities for single-cell analyses to evaluate the tumor microenvironment in clinical specimens. Sci Rep (2021) 11:341. doi: 10.1038/s41598-020-79385-w 33431933 PMC7801605

[51] Zhou JL Yang ZL Wu XR Zhang J Zhai W Chen YH. Identification of genes that correlate clear cell renal cell carcinoma and obesity and exhibit potential prognostic value. Transl Androl Urol (2021) 10:680–91. doi: 10.21037/tau-20-891 PMC7947457 33718070

[52] Mangiola S Stuchbery R Macintyre G Clarkson MJ Peters JS Costello AJ. Periprostatic fat tissue transcriptome reveals a signature diagnostic for high-risk prostate cancer. Endocr Relat Cancer (2018) 25:569–81. doi: 10.1530/ERC-18-0058 29592867

[53] Hsu HM Chu CM Chang YJ Ye JC Chen CT Jian CE. Six novel immunoglobulin genes as biomarkers for better prognosis in triple-negative breast cancer by gene co-expression network analysis. Sci Rep-Uk 9 (2019) 9:4484. doi: 10.1038/s41598-019-40826-w PMC6418134 30872752

[54] Gulli F Basile U Gragnani L Napodano C Pocino K Miele L. IgG cryoglobulinemia. Eur Rev Med Pharmacol Sci (2018) 22:6057–62. doi: 10.26355/eurrev_201809_15943 30280791

[55] Li X Chen W Yang C Huang Y Jia J Xu R. IGHG1 upregulation promoted gastric cancer malignancy *via* AKT/GSK-3beta/beta-Catenin pathway. Cancer Cell Int (2021) 21:397. doi: 10.1186/s12935-021-02098-1 34315496 PMC8314571

[56] Zhang Y Fang X Sun Y. IGHG1 promotes malignant progression in breast cancer cells through the regulation of AKT and VEGF signaling. Biomol BioMed (2023) 23:616–623. doi: 10.17305/bb.2022.8508 PMC10351095 36883223

[57] Chu J Li Y Deng Z Zhang Z Xie Q Zhang H. IGHG1 regulates prostate cancer growth *via* the MEK/ERK/c-myc pathway. BioMed Res Int (2019) 2019:7201562. doi: 10.1155/2019/7201562 31355278 PMC6637713

[58] Vitale I Manic G Coussens LM Kroemer G Galluzzi L. Macrophages and metabolism in the tumor microenvironment. Cell Metab (2019) 30:36–50. doi: 10.1016/j.cmet.2019.06.001 31269428

[59] Gabrilovich DI Ostrand-Rosenberg S Bronte V. Coordinated regulation of myeloid cells by tumours. Nat Rev Immunol (2012) 12:253–68. doi: 10.1038/nri3175 PMC3587148 22437938

[60] Chen SMY Krinsky AL Woolaver RA Wang X Chen Z Wang JH. Tumor immune microenvironment in head and neck cancers. Mol Carcinog (2020) 59:766–74. doi: 10.1002/mc.23162 PMC7282929 32017286

[61] van der Leun AM Thommen DS Schumacher TN. CD8(+) T cell states in human cancer: insights from single-cell analysis. Nat Rev Cancer (2020) 20:218–32. doi: 10.1038/s41568-019-0235-4 PMC7115982 32024970

