# Supplementary material for: Single-cell RNA sequencing reveals tumor immune microenvironment in human hypopharygeal squamous cell carcinoma and lymphatic metastasis
Source: Front Immunol. 2023 Jul 12;14:1168191. doi: 10.3389/fimmu.2023.1168191 (PMC10369788; doi:10.3389/fimmu.2023.1168191)
Supplement: Supplementary file 1 [file DataSheet_1.docx]

**
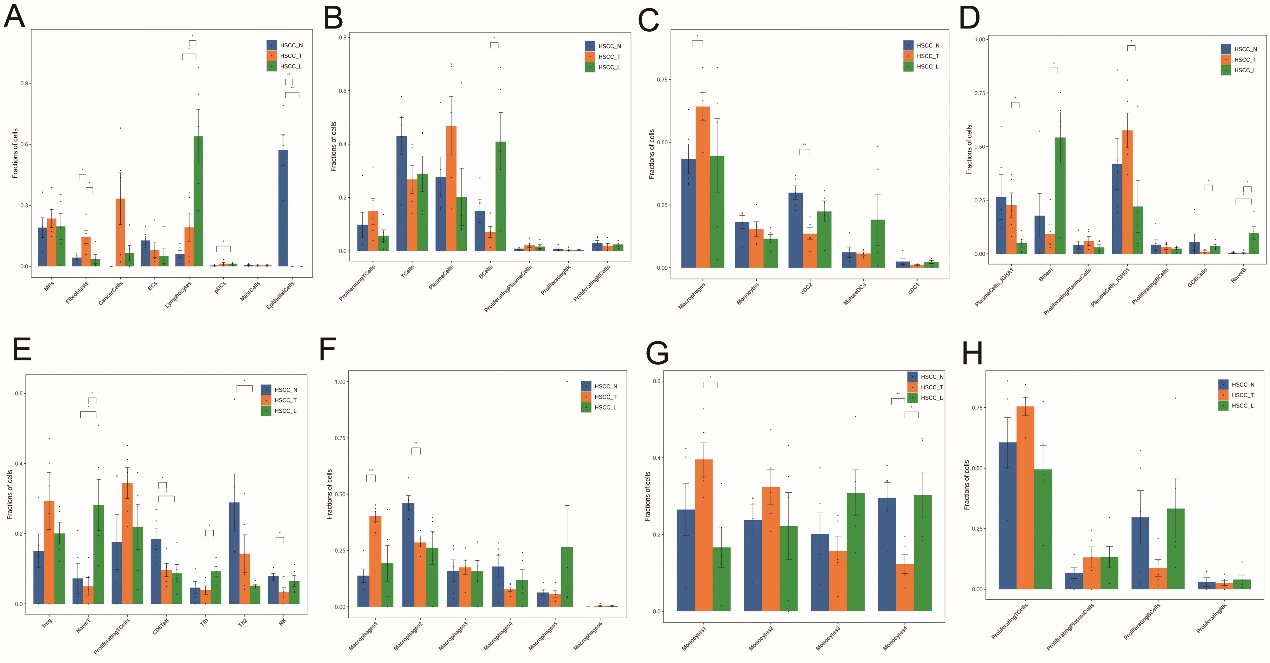
**

**SFigure1 The proportion of each cell type subdivided by cell subsets.**

1. The histogram of the difference in the proportion of cell subsets among each group. (B) The histogram of the difference in the proportion of cell subsets of lymphocytes cells among each group. (C) The histogram of the difference in the proportion of cell subsets of MPs among each group. (D) The histogram of the difference in the proportion of cell subsets among each group of B cells. (E) The histogram of the difference in the proportion of cell subsets of T cells among each group. (F) The histogram of the difference in the proportion of cell subsets of macrophages among each group. (G) The histogram of the difference in the proportion of cell subsets of monocytes among each group. (H) The histogram of the difference in the proportion of cell subsets of proliferating lymphocytes among each group.


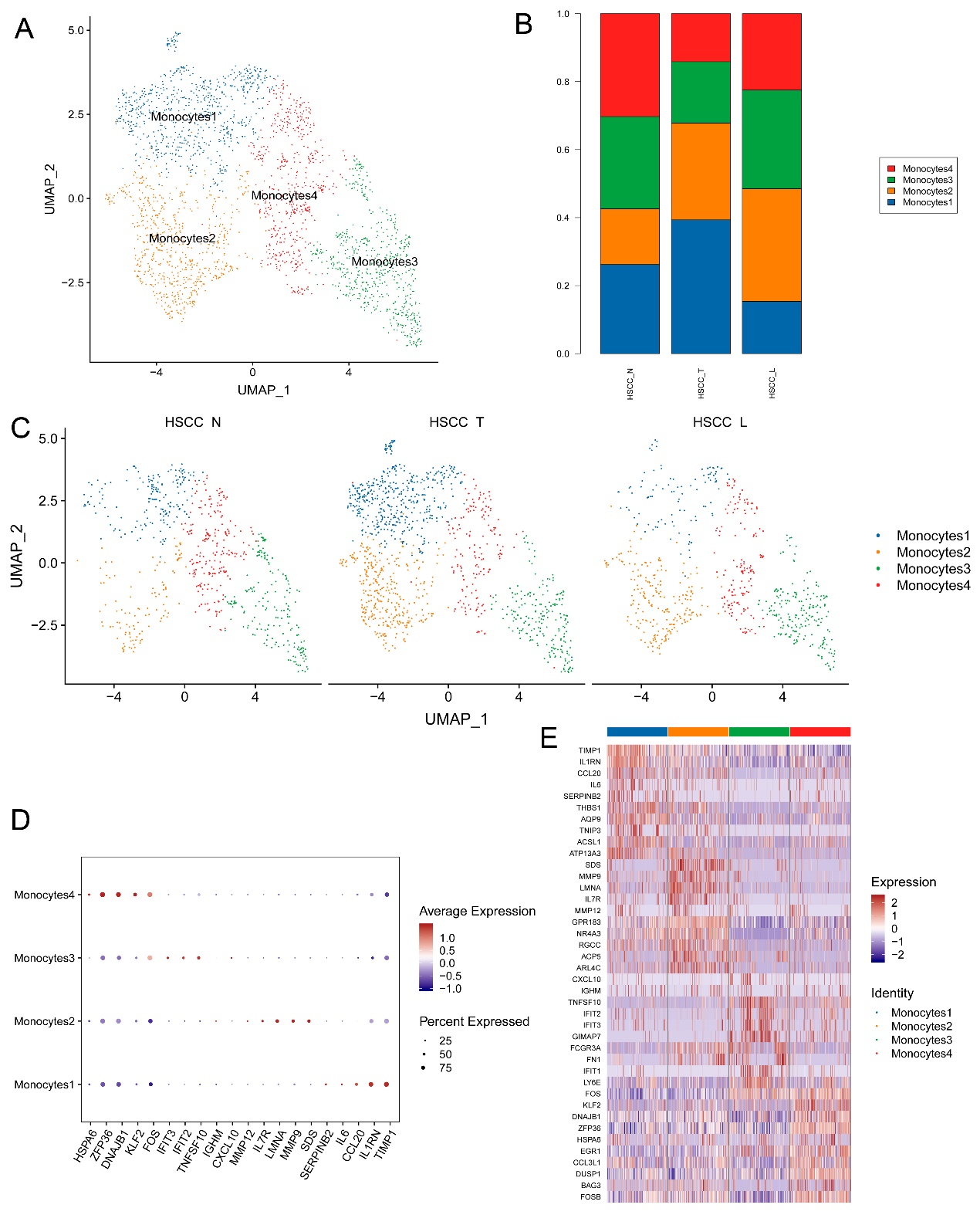


**SFigure2 Subpopulations, pseudotime trajectory and transcriptome landscape of monocytes in HSCC tissues, adjacent normal tissues and lymphatic metastasis tissues.**

1. Uniform manifold approximation and projection (UMAP) plot showing the sub classification of monocytes. (B) Bar charts showing the proportion of each monocytes subtype in HSCC tissues, adjacent normal tissues and lymphatic metastasis tissues. (C) UMAP plot of four subtypes colored in HSCC tissues, adjacent normal tissues and lymphatic metastasis tissues. (D) Bubble chart showing 5 typical genes expressed in each subtype. (E) Heatmap showing the top 10 marker genes of each subpopulation.


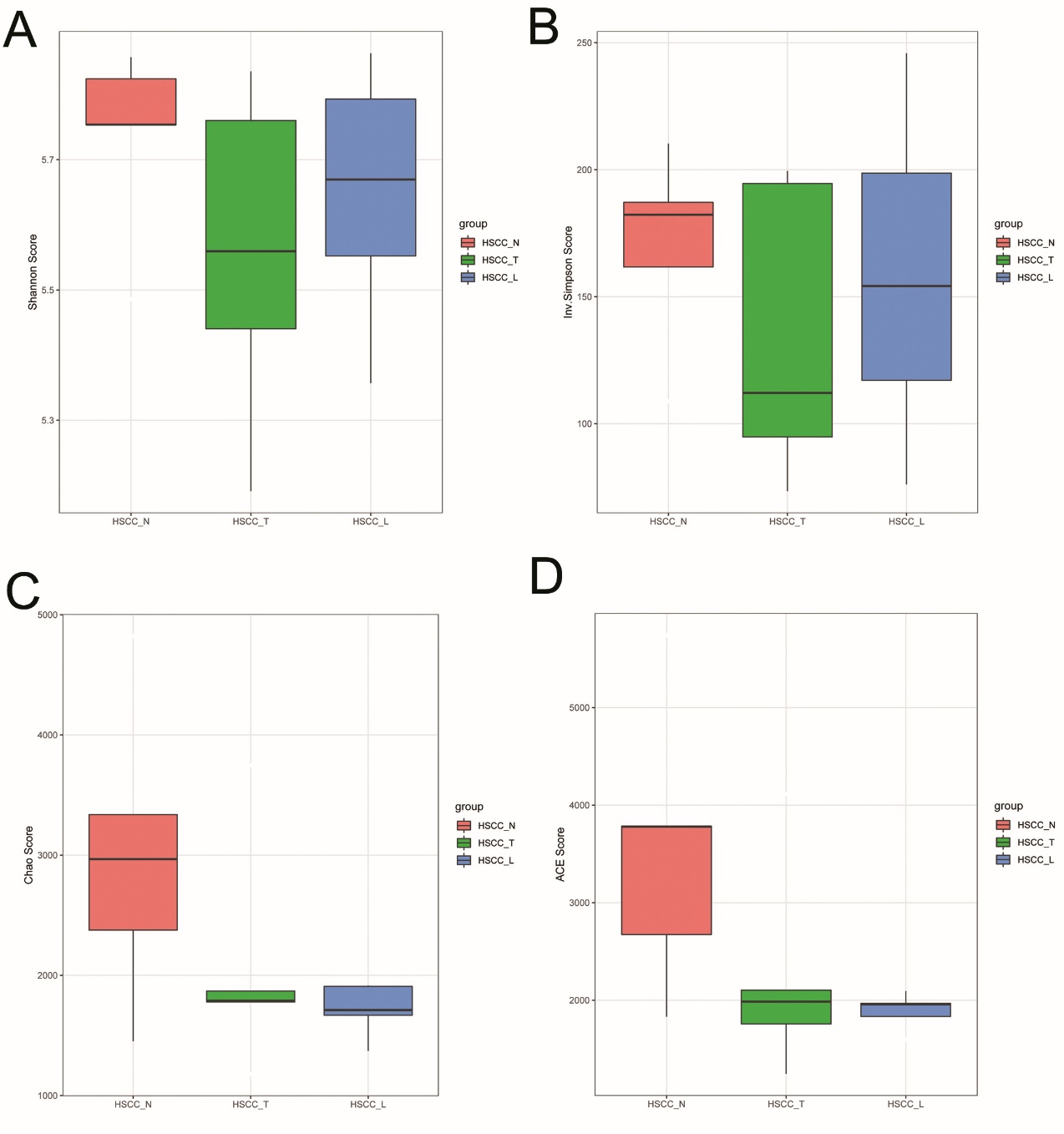


**SFigure3 TCR diversity analysis in HSCC tissues, adjacent normal tissues and lymphatic metastasis tissues.**

(A) Shannon Score of TCR diversity in HSCC tissues, adjacent normal tissues and lymphatic metastasis tissues. (B) Inv. Simpson Score of TCR diversity in HSCC tissues, adjacent normal tissues and lymphatic metastasis tissues. (C) Chao Score of TCR diversity in HSCC tissues, adjacent normal tissues and lymphatic metastasis tissues. (D) ACE Score of TCR diversity in HSCC tissues, adjacent normal tissues and lymphatic metastasis tissues.
